# Supplementary material for: Adverse Effect of Sugarcane Extract Powder (SEP) in Hyper-Lipidemic Zebrafish During a 14-Week Diet: A Comparative Analysis of Biochemical and Toxicological Efficacy Between Four SEPs and Genuine Policosanol (Raydel®)
Source: Int J Mol Sci. 2025 Sep 29;26(19):9524. doi: 10.3390/ijms26199524 (PMC12524539; doi:10.3390/ijms26199524)
Supplement: Supplementary file 1 [file ijms-26-09524-s001.zip › ijms-3892469-supplementary.pdf]

Supplementary materials

Supplementary Figure S1

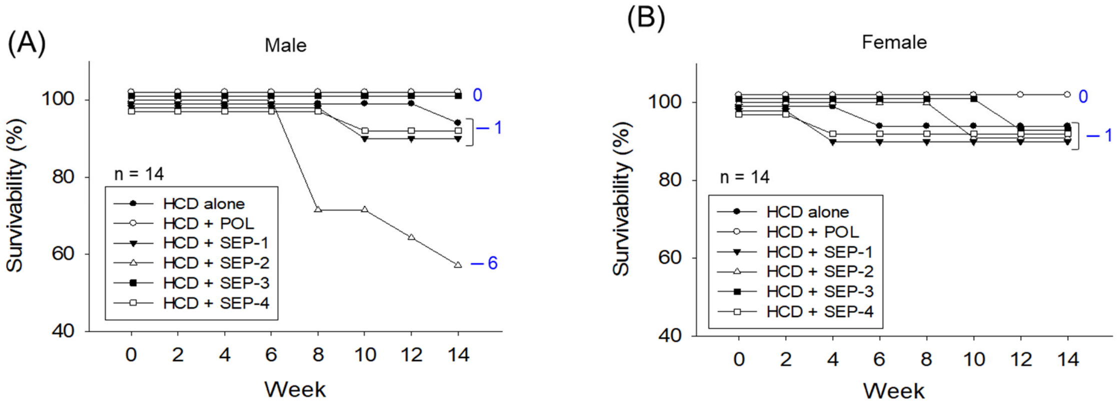

Supplementary Figure S1: Survivability kinetics of (A) male and (B) female zebrafish in HCD alone, HCD+POL, HCD+SEP-1, HCD+SEP-2, HCD+SEP-3, and HCD+SEP-4 groups during 14 weeks. The numerical value in the blue font highlights the total number dead zebrafish in the respective group at week 14. The acronyms HCD: High cholesterol diet, POL: policosanol, while SEP: sugarcane extract powder.

Supplementary images S2

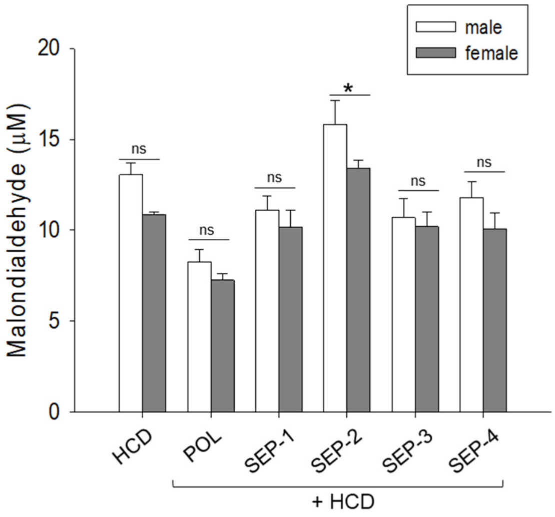

Supplementary Figure S2: A comparative malondialdehyde level in the plasma of male and female zebrafish supplemented for 14 weeks either with HCD alone, HCD+POL, HCD+SEP-1, HCD+SEP-2, HCD+SEP-3, and HCD+SEP-4. The *represent the stastistical difference at $p<0.05$; ns represents the non-significant difference between the groups. The acronyms HCD: High cholesterol diet, POL: policosanol, while SEP: sugarcane extract powder.

Supplementary Table S1: A comparative blood biochemical profile of zebrafish maintained on the normal diet (ND) that subsequently transferred to the high-cholesterol diet (HCD) for 7 weeks (pre-feeding).

Plasma biochemical parameters	Zebrafish	
	ND (14 weeks aged)	HCD (7-week consumption as pre-feeding)
Total cholesterol (TC)	130.7 ± 2.1 mg/dL	168.3 ± 12.6 mg/dL
Triglycerides (TG)	60.1 ± 1.4 mg/dL	90.6 ± 6.3 mg/dL
High density lipoprotein cholesterol (HDL-C)	70.4 ± 4.4 mg/dL	51.5 ± 3.4 mg/dL
Malondialdehyde (MDA)	4.97 ± 0.2 μM	8.2 ± 0.4 μM
Sulfhydryl content	15.2 ± 0.3 mmol/mg	12.2 ± 0.5 mmol/mg
Ferric reducing activity (FRA)	454.8 ± 10.6 μM	252.9 ± 15.4 μM
Paraoxonase activity (PON)	12.1 ± 0.2 μU/L/min	6.9 ± 0.3 μU/L/min

Section S1. Quantification of blood lipoprotein profile hepatic function biomarkers AST and ALT

The plasma total cholesterol (TC) and triglycerides (TGs) were determined using commercial assay kits (cholesterol, T-CHO, and TGs, Cleantech TS-S; Wako Pure Chemical, Osaka, Japan) as per the method suggested by the suppliers. In brief, 5 μL serum was mixed with 200 μL reaction mixture (supplied with a commercial assay kit) for the TC analysis. The content was incubated at 37°C for 10 min, resulting in a red-colored product quantified by adsorption at 490 nm (Microplate reader, Bio-Rad, Hercules, CA, USA).

Similarly, 5 μL serum was mixed with a 200 μL of TGs-specific reaction mixture (supplied with a commercial assay kit) for TGs analysis. The content was incubated for 10 min at 37°C, and the formed colored product was quantified by taking adsorption at 490 nm.

For HDL-C analysis, serum was mixed in an equal ratio with the separation solution (supplied with a commercial assay kit), followed by centrifugation at 3,000 rpm for 10 min. The supernatant (20 μL) was collected and blended with a 200 μL reaction mixture (supplied with a commercial assay kit). After 10 min incubation at 37°C, red color intensity corresponding to HDL-C was quantified by taking absorption at 490 nm.

The commercial diagnostic kit (Asan Pharmaceutical, Hwasung, Republic of Korea) was used to quantify aspartate transaminase (AST) and alanine transaminase (ALT) levels in the plasma, following the instructions suggested by the manufacturers. Briefly, 5 μL of plasma was combined with 250 μL of either AST or ALT-specific solution, as supplied in the diagnostic kit. Following a 30 min incubation for AST or 60 min incubation of ALT at 37°C, the mixture was then blended with 250 μL of the respective coloring reagent (AST or ATL-specific, provided in the diagnostic kit). After a subsequent 20 min incubation at room temperature (RT), 250 μL of 0.4 N NaOH was introduced to halt the reaction. Finally, the AST and ATL were quantified by measuring absorbance at 490 nm.

Section S2. Paraoxonase and FRA activity

Briefly, 40 μL of the plasma (1 mg/mL equivalent protein) was suspended in the 180 μL of paraoxon ethyl (0.15 g/mL) and content was incubated at RT for 120 min. The absorbance 415 nm was determined and the paraoxonase activity was expressed as μU/L/min using the molar absorbance coefficient (ε=17×10^3 M^{-1}cm^{-1}) of p-nitrophenol, a product formed by the action of paraoxonase.

To assess ferric ion reduction (FRA) capacity, 20 μL of the plasma (1 mg/mL equivalent protein) was mixed with 180 μL of FRA reagent (prepared by blending 10 mL of acetate buffer (0.2M, pH 3.6) with 1.25 mL each of 2,4,6-tripridyl-S triazin (10 mM) and $FeCl_3$ (20 mM). After incubating the mixture at RT for 60 min, absorbance was measured at 593 nm. The results were quantified in μM ferric equivalents based on a ferrous sulfate standard curve.
